# Capture Point-Based Controller Using Real-Time Zero Moment Point Manipulation for Stable Bipedal Walking in Human Environment

**DOI:** 10.3390/s19153407

**Published:** 2019-08-03

**Authors:** Young-Dae Hong

**Affiliations:** Department of Electrical and Computer Engineering, Ajou University, Suwon 16499, Korea; ydhong@ajou.ac.kr

**Keywords:** walking stability control, capture point (CP), zero moment point (ZMP) manipulation, modifiable walking pattern, 3-D linear inverted pendulum model (LIPM)

## Abstract

For collaboration of humans and bipedal robots in human environments, this paper proposes a stability control method for dynamically modifiable bipedal walking using a capture point (CP) tracking controller. A reasonable reference CP trajectory for the CP tracking control is generated using the real-time zero moment point (ZMP) manipulation without information on future footstep commands. This trajectory can be modified at any time during the single support phase according to a given footstep command. Accordingly, this makes it possible for the robot to walk stably with dynamically modifiable walking patterns, including sudden changes in navigational commands during the single support phase. A reference CP trajectory during the double support phase is also generated for continuity. The CP of the robot is controlled to track the reference trajectory using a ZMP-based CP tracking controller. The ZMP while walking is measured by the force-sensing resistor sensors mounted on the sole of each foot. A handling method for infeasible footstep commands is utilized so that the manipulated ZMP satisfies the allowable ZMP region for stability. The validity of the proposed method is verified through simulations and experiments.

## 1. Introduction

Research on bipedal robots primarily focuses on achieving stable walking. Various approaches have been developed for stable walking pattern generation and control. One such approach generated a walking pattern by representing a bipedal robot as a simple three-dimensional (3-D) linear inverted pendulum model (LIPM) [1,2]. This method used the relationship between the center of mass (COM) of the robot and the zero moment point (ZMP) to generate a stable walking pattern. Based on the 3-D LIPM, walking pattern generation methods have been developed using specific forms of ZMP trajectories to improve the walking abilities of robots [3,4,5]. Some approaches have generated walking patterns using ZMP manipulation to achieve real-time modifiable bipedal walking [6,7,8].

Achieving stable bipedal walking based on a generated walking pattern requires a stability controller. An optimal controller was introduced to track a reference ZMP trajectory using preview data [9]. This controller was then extended to an auxiliary ZMP controller for walking on uneven terrain [10], as well as a model predictive controller that compensates for strong perturbation [11]. Subsequently, walking stability control methods based on a capture point (CP) were developed. The divergent component of COM motion is defined as the CP [12,13], meaning stable walking can be achieved by controlling the CP. A CP tracking controller based on the 3-D LIPM was proposed for walking stability control [14]. In this method, a ZMP-based CP tracking control method is utilized. A reference CP trajectory for the control is generated by heuristically setting the desired CP position at the end of a step. It was extended to a model containing vertical COM motion with angular momentum to improve control performance [15,16]. The reference CP trajectory generation by a backward calculation from the next three steps was also introduced. Furthermore, a walking controller based on divergent component of motion (DCM), which means CP in 3-D, was proposed for stable bipedal walking in 3-D environments [17].

Meanwhile, to achieve robust mobility in complex environments with dynamic obstacles, a bipedal robot should have the ability to modify its walking pattern dynamically in real-time. This ability enables a robot to respond to navigational commands that are changed in real-time from footstep planners [18,19]. For dynamically modifiable bipedal walking, the COM of the robot should be able to accelerate or decelerate freely during the single support phase without any restriction of its movement. A modifiable walking pattern generator (MWPG) was developed to facilitate independent changes in COM positions and velocities at any time using real-time ZMP manipulation [6,7,8]. Consequently, the real-time generation of modifiable walking patterns was realized. However, a walking stability controller is required to ensure the stability of dynamically modifiable bipedal walking.

This paper proposes a stability control method for dynamically modifiable bipedal walking using a CP tracking controller. For CP tracking controller, a reference CP trajectory is required. In the conventional CP tracking controllers, however, the reference CP trajectory is determined heuristically or determined by the iterative method to require information on command lists of several future footsteps, which makes it difficult to immediately respond to sudden changes in navigational commands during the single support phase. In this paper, these problems are solved by generating the reference CP trajectory using the real-time ZMP manipulation from the MWPG. Namely, the reference CP trajectory for stability control of dynamically modifiable walking can be generated in real-time without information on future footstep commands. This makes it possible for the robot to walk stably with the dynamically modifiable walking patterns, including sudden changes in navigational commands during the single support phase.

A command state is defined as a navigational command set of walking periods, step lengths, and walking rotations. The MWPG generates a modifiable walking pattern to achieve a given command state through ZMP manipulation. A reference CP trajectory for walking stability control is then generated using the real-time ZMP manipulation. This trajectory can be modified at any time during the single support phase according to the given command state, because it utilizes the real-time ZMP manipulation. Additionally, a reference CP trajectory in the double support phase is generated to facilitate a continuous CP trajectory. The CP of the robot is then controlled to track the generated reference CP trajectory. Occasionally, the manipulated ZMP may move outside of the stable ZMP region, which is within the foot region. In such cases, the walking pattern that satisfies the given command state causes unstable walking, meaning the command state is infeasible for the MWPG. Therefore, a handling method for the infeasible command states is utilized so that the manipulated ZMP satisfies the allowable ZMP region for stability. The proposed method is implemented with a small-sized bipedal robot, DARwIn-OP, and the validity of the proposed method is verified through simulations and experiments.

The remainder of this paper is organized as follows. Section 2 introduces the MWPG, and COM motion according to the command state is described, along with ZMP manipulation. Section 3 proposes the CP-based stability control method for dynamically modifiable walking. CP dynamics are briefly reviewed, and the generation of a reference CP trajectory in the single support phase using real-time ZMP manipulation is described. The generation of a reference CP trajectory in the double support phase and CP tracking control are also explained. The handling method for infeasible command states is described. Finally, the overall procedure of the proposed method is presented. Simulation and experimental results are provided and discussed in Section 4, and the paper is concluded in Section 5.

## 2. Modifiable Walking Pattern Generator

### 2.1. COM Motion of 3-D LIPM

**Figure 1:**
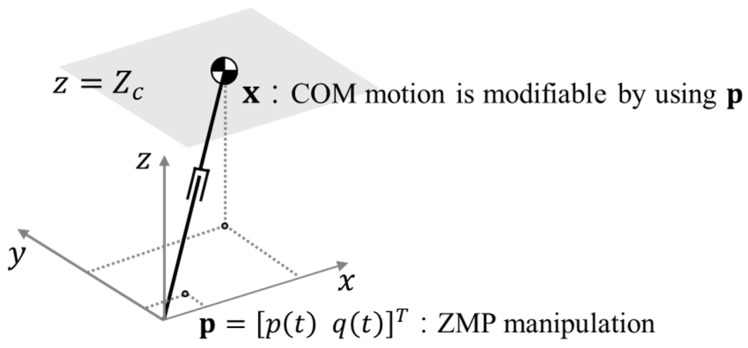
Three-dimensional (3-D) linear inverted pendulum model (LIPM) with zero moment point (ZMP) manipulation.

**Figure 2:**
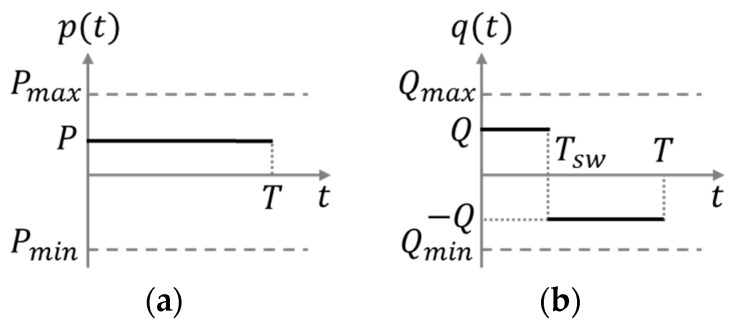
ZMP functions. (**a**) Constant function for sagittal center of mass (COM) motion. (**b**) Step function for lateral COM motion.

A bipedal robot can be modeled as a 3-D LIPM, as shown in Figure 1. Its dynamic equation is written as follows:

(1) $$\ddot{x}=\frac{x-p}{T_{c}^{2}}$$

where $x={\left[x\;y\right]}^{T}$ and $p={\left[p_{x}\;p_{y}\right]}^{T}$ are the COM and the ZMP positions for sagittal and lateral motion, respectively. $T_{c}=\sqrt{Z_{c}/g}$ is the time constant, and $Z_{c}$ is the constant height of the 3-D LIPM. The sagittal and the lateral COM motions are derived from the dynamic equation of the 3-D LIPM, and the vertical COM motion is not considered to decouple the sagittal and the lateral COM motion equations. From the solution of Equation (1), the COM equation for the 3-D LIPM is obtained as follows [6,7,8]:

(2) $$\begin{array}{c} x_{ws}\left(t\right)=A\left(t\right)x_{ws0}+b_{p}\left(t\right)\\ y_{ws}\left(t\right)=A\left(t\right)y_{ws0}+b_{q}\left(t\right) \end{array}$$

with

$$A\left(t\right)=\left[\begin{array}{cc} c\left(t\right) & s\left(t\right)\\ s\left(t\right) & c\left(t\right) \end{array}\right]\;b_{p}\left(t\right)=-\frac{1}{T_{c}}\left[\begin{array}{c} \left(s*p\right)\left(t\right)\\ \left(c*p\right)\left(t\right) \end{array}\right],\;b_{q}\left(t\right)=-\frac{1}{T_{c}}\left[\begin{array}{c} \left(s*q\right)\left(t\right)\\ \left(c*q\right)\left(t\right) \end{array}\right]$$

where $x_{ws}={\left[x\;T_{c}\dot{x}\right]}^{T}$ and $y_{ws}={\left[y\;T_{c}\dot{y}\right]}^{T}$ represent the walking state of the COM, which is defined by the COM position and the velocity in the sagittal and the lateral planes. $x_{ws0}={\left[x_{0}\;T_{c}{\dot{x}}_{0}\right]}^{T}$ and $y_{ws0}={\left[y_{0}\;T_{c}{\dot{y}}_{0}\right]}^{T}$ are the initial conditions of the walking state. c(t) and s(t) are abbreviations for $\cos h\left(t/T_{c}\right)$ and $\sin h\left(t/T_{c}\right)$, respectively. ∗ denotes the convolution operator. p(t) and q(t) are the ZMP functions for sagittal and lateral COM motions, respectively, which are used for the real-time ZMP manipulation. If the ZMP is fixed on the center of the supporting foot, namely, $p={\left[0\;0\right]}^{T}$, in Equation (1), only the homogeneous solution part of Equation (2) is utilized, which results in predetermined and unmodifiable COM motion during the single support phase. Consequently, the COM of the robot is unable to accelerate or decelerate freely during the single support phase; accordingly, it is impossible to change walking period, step length, and walking rotation independently during the single support phase. To solve this problem, the ZMP functions, p(t) and q(t), are defined in the MWPG as the constant function for sagittal COM motion and the step function for lateral COM motion, respectively, as follows:

(3) $$\begin{array}{l} p\left(t\right)=\{\begin{array}{cc} P, & \mathrm{if}\;0\;\leq t\;<T\\ 0, & \mathrm{otherwise} \end{array}\\ q\left(t\right)=\{\begin{array}{cc} Q, & \mathrm{if}\;0\leq t<T_{sw}\\ -Q, & \mathrm{if}\;T_{sw}\leq t<T \end{array} \end{array}$$

where P and Q denote the magnitudes of the ZMP functions, T denotes the remaining single support time, and $T_{sw}$ denotes the switching time for the step function. Using these ZMP functions, real-time ZMP manipulation is facilitated during the single support phase. Namely, the COM motion during the single support phase is modifiable by using $p={\left[p\left(t\right)\;q\left(t\right)\right]}^{T}$ in Equation (1) to satisfy the changes of walking period, step length, and walking rotation. The parameters for the ZMP manipulation, P, Q, T, and $T_{sw}$, are calculated from Equation (2) [6,7,8]. Consequently, the COM position and the velocity can be independently changed at any time, which enables the real-time generation of dynamically modifiable walking patterns. However, the manipulation of the ZMP should be restricted to the region where the stability of the generated walking patterns is guaranteed. Therefore, the allowable ZMP region for stable walking patterns is defined by $P_{max}$/$P_{min}$ and $Q_{max}$/$Q_{min}$ within the foot region, as shown in Figure 2.

### 2.2. Desired Walking State from Command State

The following command state is defined as a navigational footstep command set:

$$c={\left[T_{l}^{ss}\;T_{r}^{ss}\;T_{l}^{ds}\;T_{r}^{ds}\;F_{l}\;F_{r}\;S_{l}\;S_{r}\;\theta_{l}\;\theta_{r}\right]}^{T}$$

where $T_{l/r}^{ss}$, $T_{l/r}^{ds}$, $F_{l/r}$, $S_{l/r}$, and $\theta_{l/r}$ denote single support time, double support time, forward step length, side step length, and walking rotation angle for the left and the right legs, respectively. These parameters are defined with respect to the local coordinate frame attached on the support foot.

To generate a modifiable walking pattern, the desired walking state, represented by $x_{ws_{d}}^{\;}={\left[x_{d}\;T_{c}{\dot{x}}_{d}\right]}^{T}$ and $y_{ws_{d}}^{\;}={\left[y_{d}\;T_{c}{\dot{y}}_{d}\right]}^{T}$, is defined as the state of the COM at the end of each single support phase to satisfy the given command state. This walking state is uniquely obtained through the following equations, which can be derived from the walking configuration in the single and the double support phases [6,7,8]:

(4) $$\begin{array}{c} X_{ws_{d}}^{l}=\left[x_{ws_{d}}^{l};\;y_{ws_{d}}^{l}\right]={\left(A_{r}^{*}A_{l}^{*}-I_{4\times 4}\right)}^{-1}\left(A_{r}^{*}b_{l}^{*}+b_{r}^{*}\right)\\ X_{ws_{d}}^{r}=\left[x_{ws_{d}}^{r};\;y_{ws_{d}}^{r}\right]={\left(A_{l}^{*}A_{r}^{*}-I_{4\times 4}\right)}^{-1}\left(A_{l}^{*}b_{r}^{*}+b_{l}^{*}\right) \end{array}\;$$

with

$$A_{r/l}^{*}=\left[\begin{array}{cc} \cos\theta_{r/l} & -\sin\theta_{r/l}\\ \sin\theta_{r/l} & \cos\theta_{r/l} \end{array}\right]⊗\left\{\left[\begin{array}{cc} c\left(T_{r/l}^{ss}\right) & s\left(T_{r/l}^{ss}\right)\\ s\left(T_{r/l}^{ss}\right) & c\left(T_{r/l}^{ss}\right) \end{array}\right]\left[\begin{array}{cc} 1 & T_{l/r}^{ds}/T_{c}\\ 0 & 1 \end{array}\right]\right\}$$

$$b_{r/l}^{*}=\mathrm{vec}\left\{\left[\begin{array}{cc} c\left(T_{l/r}^{ss}\right) & s\left(T_{l/r}^{ss}\right)\\ s\left(T_{l/r}^{ss}\right) & c\left(T_{l/r}^{ss}\right) \end{array}\right]\left[\begin{array}{cc} F_{l/r} & S_{l/r}\\ 0 & 0 \end{array}\right]\left[\begin{array}{cc} \cos\theta_{l/r} & -\sin\theta_{l/r}\\ \sin\theta_{l/r} & \cos\theta_{l/r} \end{array}\right]\right\}$$

where ⊗ represents the Kronecker product, and vec(·) represents the vectorization of the matrix by stacking the columns of the matrix into a single column vector. As shown in (4), information regarding the command state is involved in the matrices, $A_{r/l}^{*}$ and $b_{r/l}^{*}$, which are used to calculate the desired walking state.

After obtaining the desired walking state, it is necessary to manipulate the ZMP for the transition from the current walking state to the desired walking state. To achieve the desired walking state, the required parameters for the ZMP functions in Equation (3), P, Q, T, and $T_{sw}$, should be determined. These parameters can be obtained using Equation (2) [6,7,8]. Using these parameters, ZMP manipulation occurs whenever the command state changes, which enables the current walking state to reach the desired state by accelerating or decelerating the COM of the robot.

## 3. CP-Based Stability Control Method for Dynamically Modifiable Walking

### 3.1. CP Dynamics

The COM motion of the 3-D LIPM can be decomposed into convergent and divergent components. The CP refers to the divergent component of COM motion and is defined as follows [12,13]:

(5) $$\xi =x+T_{c}\dot{x}\;$$

where $\xi ={\left[\xi_{x}\;\xi_{y}\right]}^{T}$ is the CP position and Equation (5) can be rewritten as follows:

(6) $$\dot{x}=\frac{\xi -x}{T_{c}}$$

Equation (6) is stable because the pole of the transfer function is $-1/T_{c}$, which indicates that the relationship between the CP and the COM is stable. Accordingly, the COM always follows the CP trajectory.

From the differentiation of Equation (5) using Equations (1) and (6), the following equation can be obtained:

(7) $$\dot{\xi }=\dot{x}+T_{c}\ddot{x}=\frac{\xi -p}{T_{c}}.\;\;\;\;$$

Equation (7) is unstable because the pole of the transfer function is $1/T_{c}$, which indicates that the relationship between the ZMP and the CP is unstable. Namely, the CP diverges from the ZMP. Therefore, a ZMP-based CP tracking controller using an appropriate CP reference trajectory is required to control the divergent components of the COM motion for stable bipedal walking.

### 3.2. Reference CP Trajectory Generation in Single Support Phase Using Real-Time ZMP Manipulation

For the CP tracking controller, a reference CP trajectory is required. It can be obtained from the solution of Equation (7) as follows:

(8) $$\xi_{ref}\left(t\right)=e^{\frac{t}{T_{c}}}\xi +\left(1-e^{\frac{t}{T_{c}}}\right)p.$$

**Figure 3:**
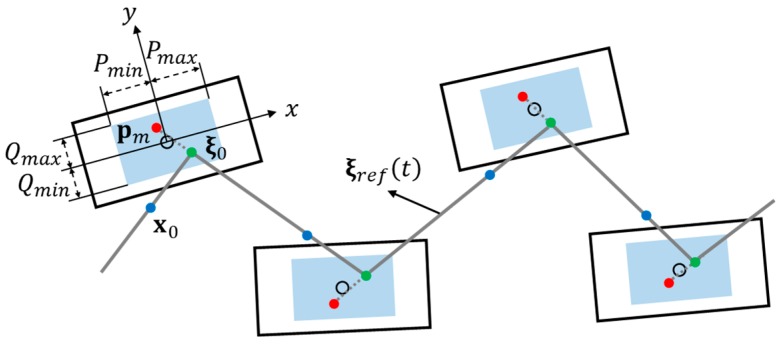
Reference capture point (CP) trajectory generation using ZMP manipulation.

In the conventional CP tracking controllers, the initial CP and ZMP positions, ξ and p, in Equation (8) for the reference CP trajectory generation, were heuristically determined by using some offset from the center of the supporting foot or determined by a backward iteration method using information on command lists of several future footsteps [14,15,16,17]. The conventional methods are able to cope with the change at each footstep of navigational command by recalculating the backward iteration method. However, it is difficult to immediately respond to sudden change of navigational command in the single support phase by using the backward iteration method, because the method requires a sequence of future footstep commands to generate the reference CP trajectory. In the proposed method, ξ and p are set by using the real-time ZMP manipulation from the MWPG without information on future footstep commands as follows:

(9) $$\xi =\xi_{0}=x_{0}^{\;}+T_{c}{\dot{x}}_{0}^{\;}$$

(10) $$p=p_{m}={\left[p_{xm}^{\;}\;p_{ym}^{\;}\right]}^{T}$$

with

$$p_{xm}^{\;}=\{\begin{array}{ll} P, & \;\mathrm{if}\;F_{l/r}^{cur}\neq F_{l/r}^{pre}\\ 0, & \;\mathrm{otherwise} \end{array}\;p_{ym}^{\;}=\{\begin{array}{ll} Q, & \;\mathrm{if}\;S_{r}^{cur}\neq S_{r}^{pre}\\ -Q, & \;\mathrm{else}\;\mathrm{if}\;S_{l}^{cur}\neq S_{l}^{pre}\\ 0, & \;\mathrm{otherwise} \end{array}$$

where $x_{0}^{\;}={\left[x_{0}^{\;}\;y_{0}^{\;}\right]}^{T}$ and ${\dot{x}}_{0}^{\;}={\left[{\dot{x}}_{0}^{\;}\;{\dot{y}}_{0}^{\;}\right]}^{T}$ denote the initial COM position and the velocity of the nominal COM trajectory, respectively. $F_{l/r}^{cur}$/$F_{l/r}^{pre}$ and $S_{l/r}^{cur}$/$S_{l/r}^{pre}$ denote the current/previous forward and the side step lengths, respectively. Figure 3 illustrates the procedure for reference CP trajectory generation using ZMP manipulation. ξ is set to $\xi_{0}$, which is the initial CP position and is calculated using Equation (5) with $x_{0}^{\;}$ and ${\dot{x}}_{0}$ from the MWPG. p is set to $p_{m}$, which is defined as a *modifiable* ZMP position and is determined by using the ZMP functions that are obtained to achieve the desired walking state derived from the given command state. This position can be changed at any time during the single support phase according to the command state. Specifically, it is modifiable within the allowable ZMP region (blue rectangle) and not fixed on the center of the supporting foot. Consequently, the reference CP trajectory can be generated in real-time through the ZMP manipulation, which facilitates real-time stability control for the dynamically modifiable walking pattern, including sudden changes in footstep commands during the single support phase.

### 3.3. Reference CP Trajectory Generation in Double Support Phase

For a continuous CP trajectory, the reference CP trajectory during the double support phase is generated through cubic spline interpolation as follows:

(11) $$\xi_{ref}\left(t\right)=a_{0}+a_{1}t+a_{2}t^{2}+a_{3}t^{3}\;\;,$$

with

$$a_{0}=\xi_{ref}\left(0\right),\;a_{1}={\dot{\xi }}_{ref}\left(0\right)$$

$$a_{2}=\frac{3\left\{\xi_{ref}\left(T_{l/r}^{ds}\right)-\xi_{ref}\left(0\right)\right\}}{T_{l/r}^{ds}{}^{2}\;}-\frac{2{\dot{\xi }}_{ref}\left(0\right)}{T_{l/r}^{ds}}-\frac{{\dot{\xi }}_{ref}\left(T_{l/r}^{ds}\right)}{T_{l/r}^{ds}},$$

$$a_{3}=-\frac{2\left\{\xi_{ref}\left(T_{l/r}^{ds}\right)-\xi_{ref}\left(0\right)\right\}}{T_{l/r}^{ds}{}^{3}\;}+\frac{\left\{{\dot{\xi }}_{ref}\left(T_{l/r}^{ds}\right)+{\dot{\xi }}_{ref}\left(0\right)\right\}}{T_{l/r}^{ds}{}^{2}\;},$$

where $\xi_{ref}\left(0\right)$ and ${\dot{\xi }}_{ref}\left(0\right)$ are obtained from the nominal COM position and the velocity at the end of the single support phase, $x_{T_{l/r}^{ss}}$ and ${\dot{x}}_{T_{l/r}^{ss}}$. Because the nominal COM trajectory during the double support phase is generated to travel with constant velocity in the MWPG, $\xi_{ref}\left(T_{l/r}^{ds}\right)$ and ${\dot{\xi }}_{ref}\left(T_{l/r}^{ds}\right)$ are obtained from the nominal COM position and the velocity at the end of the double support phase, $x_{T_{l/r}^{ds}}$ and ${\dot{x}}_{T_{l/r}^{ds}}$, as follows:

(12) $$\begin{array}{l} \xi_{ref}\left(T_{l/r}^{ds}\right)=x_{T_{l/r}^{ds}}+T_{c}{\dot{x}}_{T_{l/r}^{ds}}\\ {\dot{\xi }}_{ref}\left(T_{l/r}^{ds}\right)={\dot{x}}_{T_{l/r}^{ds}}+T_{c}{\ddot{x}}_{T_{l/r}^{ds}}={\dot{x}}_{T_{l/r}^{ds}} \end{array}$$

with

$$x_{T_{l/r}^{ds}}=x_{T_{l/r}^{ss}}+T_{l/r}^{ds}{\dot{x}}_{T_{l/r}^{ss}},\;\;\;{\dot{x}}_{T_{l/r}^{ds}}={\dot{x}}_{T_{l/r}^{ss}}.$$

### 3.4. CP Tracking Control

In order to control the current CP to track the reference CP trajectory, the following control law is derived from Equation (8) [15]:

(13) $$p_{d}\left(t\right)=\frac{1}{1-e^{\frac{\Delta T}{T_{c}}}}\xi_{t}\left(t\right)-\frac{e^{\frac{\Delta T}{T_{c}}}}{1-e^{\frac{\Delta T}{T_{c}}}}\xi_{c},$$

with

$$\xi_{t}\left(t\right)=\xi_{ref}\left(t+\Delta T\right),$$

where $\xi_{t}={\left[\xi_{xt}\;\xi_{yt}\right]}^{T}$ and $\xi_{c}={\left[\xi_{xc}\;\xi_{yc}\right]}^{T}$ are the target and the current CPs, respectively. ΔT is the sampling time for controlling the CP. $p_{d}\left(t\right)$ is the required ZMP for the current CP to track the target CP. The force for the required ZMP can be obtained using Equation (1) as follows:

(14) $$F_{d}=m\frac{x-p_{d}}{T_{c}^{2}}=\frac{mg}{Z_{c}}\left(x-p_{d}\right),$$

where m is the mass of the COM. The required acceleration of the COM for the calculated force can be derived from the current force exerted on the COM, $F_{c}$, as follows:

(15) $${\ddot{x}}_{d}=K_{f}\left(F_{d}-F_{c}\right),$$

where $K_{f}=\left[k_{xf}\;0;0\;k_{yf}\right]$ is the coefficient matrix. By inserting Equation (14) into Equation (15), the required acceleration of the COM for satisfying the desired ZMP can be rewritten as follows:

(16) $${\ddot{x}}_{d}=K_{f}\frac{mg}{Z_{c}}\left(p_{d}-p_{c}\right),$$

where $p_{c}$ is the current ZMP. The target COM position and the velocity, $x_{t}$ and ${\dot{x}}_{t}$, are calculated by adding the integration of Equation (16) to the nominal COM trajectory from (2). Consequently, they enable the ZMP while walking to follow the desired ZMP for CP tracking control.

### 3.5. Handling Infeasible Command States and the Corresponding Desired Walking States

As mentioned in Section 2, the ZMP functions, p(t) and q(t), should stay within the allowable ZMP region where the stability of the generated walking patterns is guaranteed. If P and Q of the ZMP functions are larger/smaller than $P_{max}$/$P_{min}$ and $Q_{max}$/$Q_{min}$, then the desired walking state for a given command state cannot be achieved from the current walking state by using ZMP manipulation within the allowable region. Namely, the command state and the corresponding desired walking state are infeasible in the MWPG. Therefore, a handling method for the infeasible command states and corresponding desired walking states is required for the generation of a stable walking pattern. Command states provided by a footstep planner are entered as inputs to the MWPG at every sampling time. Some of these command states may be infeasible, since the footstep planner does not consider the feasibility of the planned footstep commands. $f={\left[F_{l}\;F_{r}\;S_{l}\;S_{r}\;\theta_{l}\;\theta_{r}\right]}^{T}$ is defined as the placement information of a footstep. If an infeasible command state is given, f of the infeasible command state is modified to achieve a feasible command state through a binary search algorithm. A binary search algorithm is appropriate for real-time walking pattern generation because it has a very low computational cost.

Algorithm 1 shows the pseudo code of the handling method, where $f_{pre}$ and $f_{d}$ refer to the footstep placement information of the previous and the desired command states, $c_{pre}$ and $c_{d}$, respectively. f(·) denotes the function for calculating the desired walking state from the command state using (4). N is the total number of iterations. After initialization, the modified desired walking state, $X_{m}$, is calculated using $c_{m}$, where $f_{m}$ is the mean value of $f_{0}$ and $f_{1}$. If $X_{m}$ is feasible, then $f_{m}$ is set to $f_{0}$ and the feasible command state and the desired walking state, $c_{f}$ and $X_{ws_{d}}$, are updated. Otherwise, $f_{m}$ is set to $f_{1}$. These steps are repeated until the current iteration number, n, reaches N. If a feasible command state is not obtained before n reaches N, the previous command state is used instead. The ZMP manipulation is not required for the same command state as the previous command state. Therefore, stable walking is guaranteed.

**Algorithm 1.** Handling infeasible command states and desired walking states.  /* initialization */  $f_{0}\leftarrow f_{pre};$  $f_{1}\leftarrow f_{d};$  $c_{f}\leftarrow c_{pre};$  $X_{ws_{d}}\leftarrow f\left(c_{f}\right);$  n←0;  /* start binary search algorithm */  **while**
n<N
**do**   $f_{m}\leftarrow \left(f_{0}+f_{1}\right)/2;$   $X_{m}\leftarrow f\left(c_{m}\right);$   **if**
$X_{m}$ is *feasible*
**then**    $f_{0}\leftarrow f_{m};$    $c_{f}\leftarrow c_{m};$    $X_{ws_{d}}\leftarrow X_{m};$    break;   **else**    $f_{1}\leftarrow f_{m};$   **end if**   n←n+1;  **end while**

### 3.6. Overall Procedure

**Figure 4:**
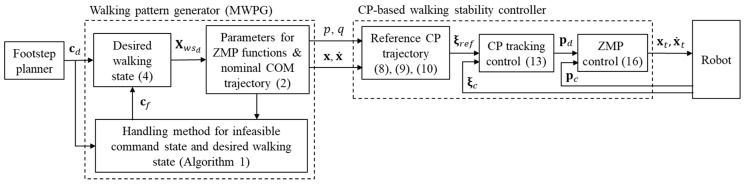
Overall procedure of the proposed method.

Figure 4 illustrates the overall procedure of the proposed method. The desired command state, $c_{d}$, provided by a footstep planner is entered as an input to the proposed algorithm at every sampling time. The desired walking state, $X_{ws_{d}}$, is obtained from $c_{d}$ using Equation (4). The parameters of the ZMP functions, P, Q, T, and $T_{sw}$, are calculated to achieve $X_{ws_{d}}$ for the current walking state. The nominal COM trajectory, x/$\dot{x}$, is calculated using Equation (2) with the ZMP functions, p(t) and q(t), and the current walking state is updated. The feasibility of $c_{d}$ and $X_{ws_{d}}$ is determined by the allowable ZMP region, $P_{max}$/$P_{min}$ and $Q_{max}$/$Q_{min}$. If the given $c_{d}$ is infeasible, it is substituted with a feasible command state, $c_{f}$, obtained by Algorithm 1, and the corresponding desired walking state and the ZMP function parameters are recalculated. The reference CP trajectory in the single support phase, $\xi_{ref}$, is generated using Equations (8)–(10). Note that $\xi_{ref}$ in the double support phase is generated using Equations (11) and (12). The desired ZMP, $p_{d}$, is calculated using Equation (13) with $\xi_{ref}$ and the current CP, $\xi_{c}$. $p_{d}$ is then transferred to the ZMP controller, which generates the target COM position/velocity, $x_{t}$**/**${\dot{x}}_{t}$, using Equation (16) with the current ZMP, $p_{c}$. Subsequently, the trajectories for every leg joint of the robot are calculated by inverse kinematics. Note that the closed-form solution for inverse kinematics is derived by using algebra and geometry in the leg configuration of the bipedal robot. Therefore, the unique angles for every leg joint are obtained by the inverse kinematics.

## 4. Experimental Results and Discussion

The proposed method was implemented for a small-sized bipedal robot, DARwIn-OP. The height and the weight of the robot are 45.5 cm and 2.8 kg, respectively. DARwIn-OP has twenty degrees of freedom (DOFs) with Dynamixel MX-28 servo actuators (two DOFs for the head, six for the arms, and twelve for the legs). It uses an Intel Atom Z530 central processing unit (1.6 GHz) as the main controller, which calculates the proposed algorithm in real-time every 5 ms. Four force-sensing resistors (FSRs) are mounted on the sole of each foot to measure ground reaction forces (GRFs) and ZMPs while walking. The measurement range of the FSR is from 0.493 N to 65.535 N, and the unit of measurement is 0.001 N. The ZMP is calculated as follows:

(17) $$p=\frac{\sum_{i=1}^{4}\left(f_{li}p_{li}+f_{ri}p_{ri}\right)}{\sum_{i=1}^{4}\left(f_{li}+f_{ri}\right)}$$

where $p_{li}$ and $p_{ri}$ (i=1, 2, 3, and 4) denote the 2 × 1 position vectors of the FSRs of the left and the right feet, respectively. $f_{li}$ and $f_{ri}$ denote the GRF measured by each FSR. Note that the proposed method is not limited to the application to DARwIn-OP.

### 4.1. Simulation Results

The simulations were performed using the DARwIn-OP simulation model by Webots, which is a 3-D dynamic robotics simulator [20]. Four FSRs were equipped on the sole of each foot in the DARwIn-OP simulation model to measure the ZMP in the simulations. The following footstep commands were realized for the simulation of modifiable bipedal walking, including a sudden adjustment of foot placement during the single support phase in which step lengths were independently changed while maintaining the same walking period for each footstep. The walking rotation angles were set to zero to verify the inherent performances of the proposed method without the influence of disturbances such as slip:

(1) Initial command state, $c={\left[0.4\;0.4\;0.4\;0.4\;4.0\;4.0\;7.4\;-7.4\;0.0\;0.0\right]}^{T}$
(2) After 2nd step, $c={\left[0.4\;0.4\;0.4\;0.4\;8.0\;8.0\;6.4\;-7.4\;0.0\;0.0\right]}^{T}$
  (2-1) After t=0.2 s at 3^rd^ step, $c={\left[0.4\;0.4\;0.4\;0.4\;3.0\;3.0\;6.4\;-7.4\;0.0\;0.0\right]}^{T}$
(3) After 3rd step, $c={\left[0.4\;0.4\;0.4\;0.4\;8.0\;8.0\;6.4\;-10.4\;0.0\;0.0\right]}^{T}$
(4) After 4th step, $c={\left[0.4\;0.4\;0.4\;0.4\;4.0\;4.0\;7.4\;-10.4\;0.0\;0.0\right]}^{T}$
(5) After 5th step, $c={\left[0.4\;0.4\;0.4\;0.4\;1.0\;1.0\;7.4\;-10.4\;0.0\;0.0\right]}^{T}$
(6) After 6th step, $c={\left[0.4\;0.4\;0.4\;0.4\;2.0\;2.0\;7.4\;-10.4\;0.0\;0.0\right]}^{T}$
(7) After 7th step, $c={\left[0.4\;0.4\;0.4\;0.4\;1.0\;1.0\;7.4\;-8.4\;0.0\;0.0\right]}^{T}$
(8) After 8th step, $c={\left[0.4\;0.4\;0.4\;0.4\;-3.0\;-3.0\;8.4\;-8.4\;0.0\;0.0\right]}^{T}$
(9) After 9th step, $c={\left[0.4\;0.4\;0.4\;0.4\;-5.0\;-5.0\;8.4\;-7.4\;0.0\;0.0\right]}^{T}$
(10) After 10th step, $c={\left[0.4\;0.4\;0.4\;0.4\;0.0\;0.0\;7.4\;-7.4\;0.0\;0.0\right]}^{T}$

where time, length, and angle units are given in seconds, centimeters, and degrees, respectively.

**Figure 5:**
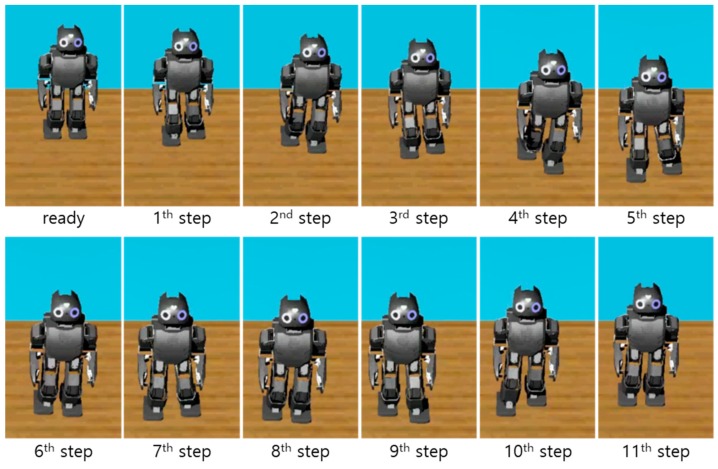
Snapshots of the first simulation result with the proposed CP-based walking stability controller (left to right, top to bottom).

**Figure 6:**
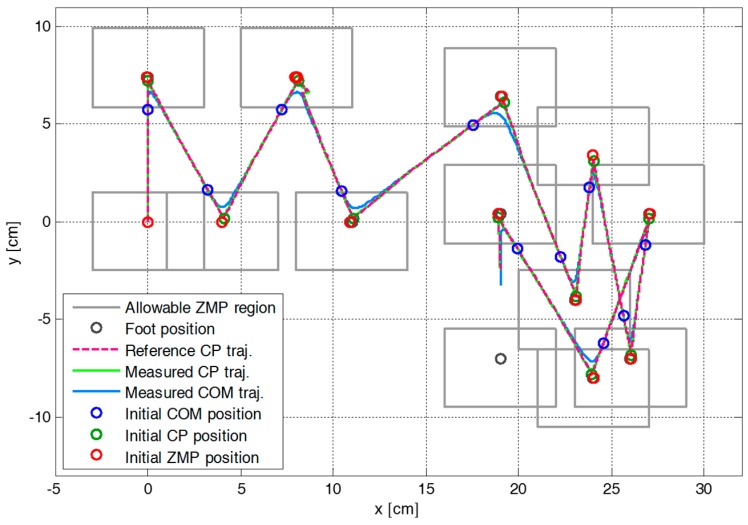
Reference CP trajectory, $\xi_{ref}$, generated through ZMP manipulation, as well as measured CP and COM trajectories, $\xi_{c}$ and $x_{c}$, for the modifiable walking pattern in the first simulation.

Figure 5 shows the snapshots of the first simulation result with the proposed CP-based walking stability controller, and Figure 6 illustrates the reference CP trajectory, $\xi_{ref}$, generated through ZMP manipulation, as well as the measured CP and COM trajectories, $\xi_{c}$ and $x_{c}$, for the modifiable walking pattern in the first simulation. The initial CP position at each footstep (small green circle), $\xi_{0}$, was derived from the initial nominal COM position (small blue circle) and the velocity in the single support phase. The modifiable ZMP position (small red circle), $p_{m}$, was derived from the ZMP functions for the given footstep command, which fell within the allowable ZMP region (thin rectangle) set as $P_{max}$ = 3.0 cm, $P_{min}$ = −3.0 cm, $Q_{max}$ = 2.5 cm, and $Q_{min}$ = −1.5 cm.

**Figure 7:**
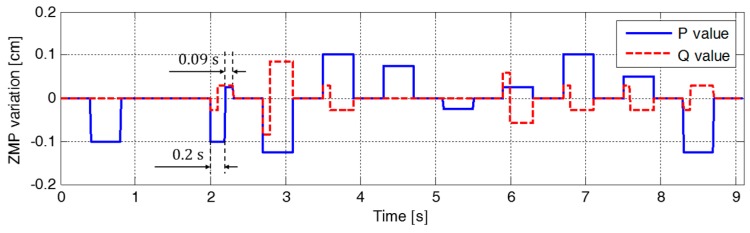
Variation in the parameters of the ZMP functions in the first simulation.

**Figure 8:**
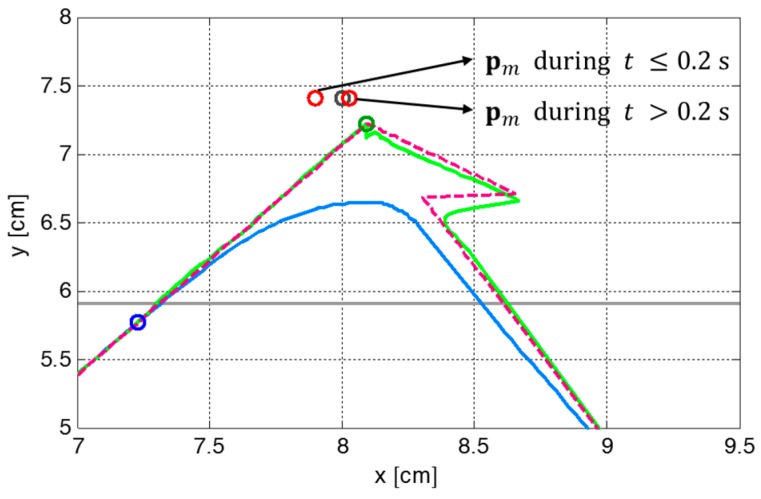
Variation in the modifiable ZMP position, $p_{m}$, as well as CP and COM trajectories, $\xi_{ref}$, $\xi_{c}$, and $x_{c}$, when the command state for the third footstep changed during the single support phase.

Figure 7 illustrates the variation in the parameters of the ZMP functions relative to the center of the supporting foot. The figure indicates that ZMP manipulation occurred whenever the footstep command changed. Therefore, the modifiable ZMP positions also changed through the ZMP manipulation. Specifically, the forward step length of the third footstep was modified from 8.0 cm to 3.0 cm at a time 0.2 s after the start of the single support phase of 0.4 s. The footstep command was modified in the single support phase, which resulted in ZMP manipulation. Figure 7 shows that the ZMP function for sagittal COM motion, p(t), was modified at t=2.2 s. For the abruptly shortened step length, the parameter of the ZMP function, P, was changed, and the remaining single support time, T, was modified from 0.2 s to 0.09 s. From the modified ZMP function, the modifiable ZMP position was also changed to realize the modified footstep command. Figure 8 presents an enlarged view of the area around the second footstep in Figure 6. The modifiable ZMP position for the third footstep was changed to a more forward position compared to that for the forward step length before modification. Consequently, the reference CP trajectory was modified in real-time according to the changed modifiable ZMP position. The real CP and COM trajectories were also adjusted to track the modified reference CP trajectory, as shown in Figure 8.

**Figure 9:**
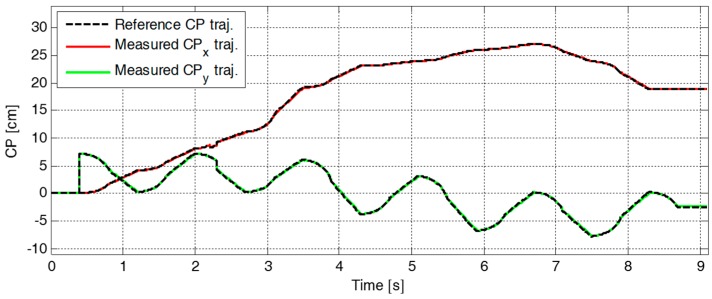
Reference and measured CP trajectories, $\xi_{ref}$ and $\xi_{c}$, in the first simulation.

**Figure 10:**
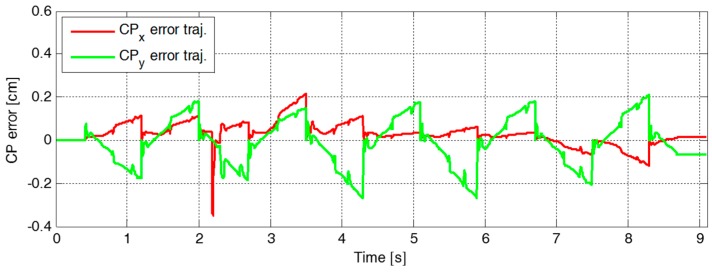
Error trajectories between the reference and the measured CPs, $\xi_{ref}$ and $\xi_{c}$, in the first simulation.

**Figure 11:**
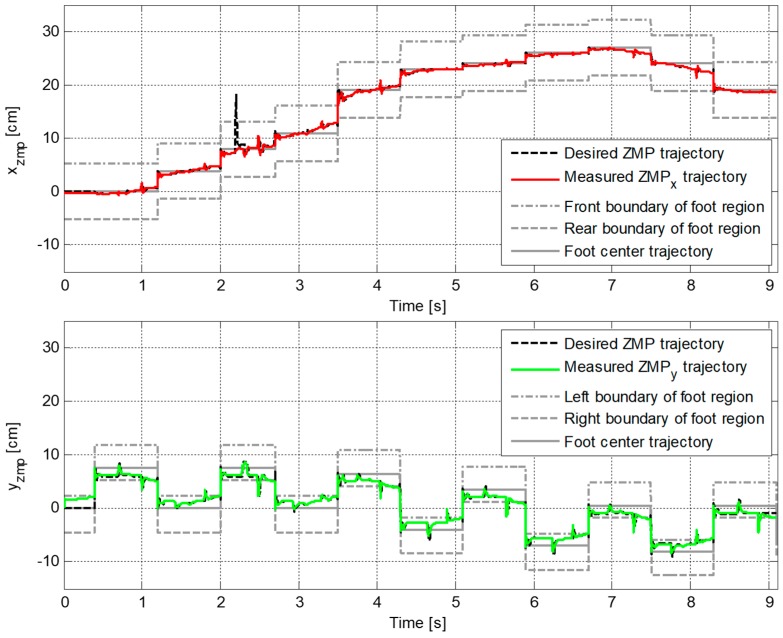
Trajectories of desired ZMP for CP tracking control and measured ZMP, $p_{d}$ and $p_{c}$, in the first simulation.

Figure 9 plots the reference and the measured CP trajectories in the *x*-axis and the *y*-axis. The real CP trajectory followed the reference trajectory with small error, as shown in Figure 10, which was facilitated by the CP tracking controller that enabled the current ZMP to follow the desired ZMP trajectory. In Figure 11, there is a peak in the desired ZMP trajectory for CP tracking control in the *x*-axis at t=2.2 s. This peak was caused by the instant variation in the target CP resulting from the change in the footstep command. However, the following desired ZMP trajectory was generated normally, meaning the ZMP followed the desired trajectory continuously without being influenced by the peak. It can be seen that the ZMP was measured with a small variation from the foot center trajectory. Consequently, the real CP trajectory was able to successfully follow the reference CP trajectory for stable modifiable bipedal walking.

To demonstrate the effectiveness of the infeasible command state handling method, an additional simulation was performed with the following footstep commands:

(1) Initial command state, $c={\left[0.4\;0.4\;0.4\;0.4\;-9.0\;-9.0\;7.4\;-7.4\;0.0\;0.0\right]}^{T}$
(2) After 1st step, $c={\left[0.4\;0.4\;0.4\;0.4\;-9.0\;-9.0\;7.4\;-10.4\;0.0\;0.0\right]}^{T}$
(3) After 2nd step, $c={\left[0.4\;0.4\;0.4\;0.4\;5.0\;5.0\;7.4\;-10.4\;0.0\;0.0\right]}^{T}$
(4) After 3rd step, $c={\left[0.4\;0.4\;0.4\;0.4\;5.0\;5.0\;7.4\;-7.4\;0.0\;0.0\right]}^{T}$
(5) After 5th step, $c={\left[0.4\;0.4\;0.4\;0.4\;0.0\;0.0\;7.4\;-7.4\;0.0\;0.0\right]}^{T}$

DARwIn-OP has a relatively large foot for its leg length, which makes most footstep commands feasible because of its relatively large allowable ZMP region. Therefore, in this simulation, the allowable ZMP region was intentionally reduced to $P_{max}$ = 0.3 cm, $P_{min}$ = −0.3 cm, $Q_{max}$ = 0.25 cm, and $Q_{min}$ = −0.15 cm to simulate infeasible command state handling.

**Figure 12:**
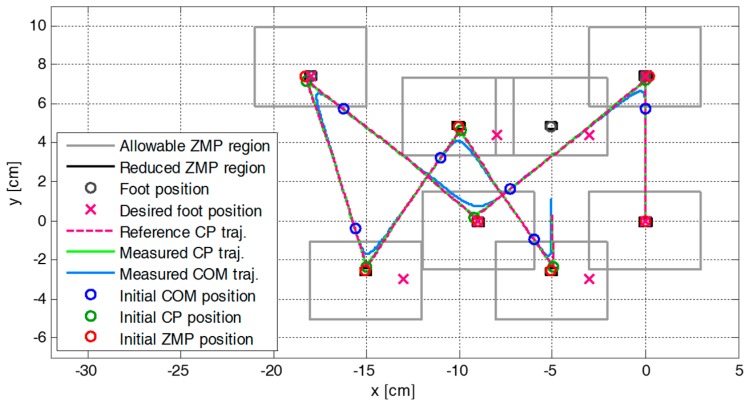
Reference CP trajectory, $\xi_{ref}$, generated through ZMP manipulation, as well as the measured CP and COM trajectories, $\xi_{c}$ and $x_{c}$, for the modifiable walking pattern with an infeasible command state in the second simulation.

**Figure 13:**
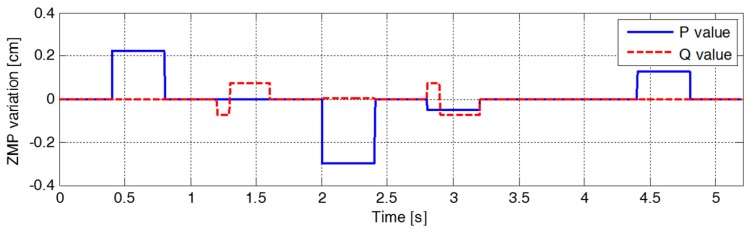
Variation in parameters for ZMP functions in the second simulation.

Figure 12 shows that the robot initially walked straight backward and then changed its walking rotation after the second footstep to move forward with a modified forward step length of 5.0 cm and side step length of −10.4 cm. At this moment, ZMP manipulation was required to achieve the desired walking state for the modified footstep command. However, the ZMP required by the manipulation fell outside of the reduced allowable ZMP region (small thick rectangle), meaning that the footstep command was infeasible. Therefore, the handling method modified the infeasible footstep command (pink cross) into a feasible footstep command for the third footstep (small black circle) with a forward step length of 2.9 cm and side step length of −9.9 cm. Accordingly, the parameters for the ZMP functions were modified to fall within the reduced allowable ZMP region, as shown in Figure 13. Consequently, a reference CP trajectory was generated through ZMP manipulation within the allowable region, and stable walking control was achieved. After the third footstep, the robot walked stably according to the given (feasible) footstep commands.

### 4.2. Experimental Results

**Figure 14:**
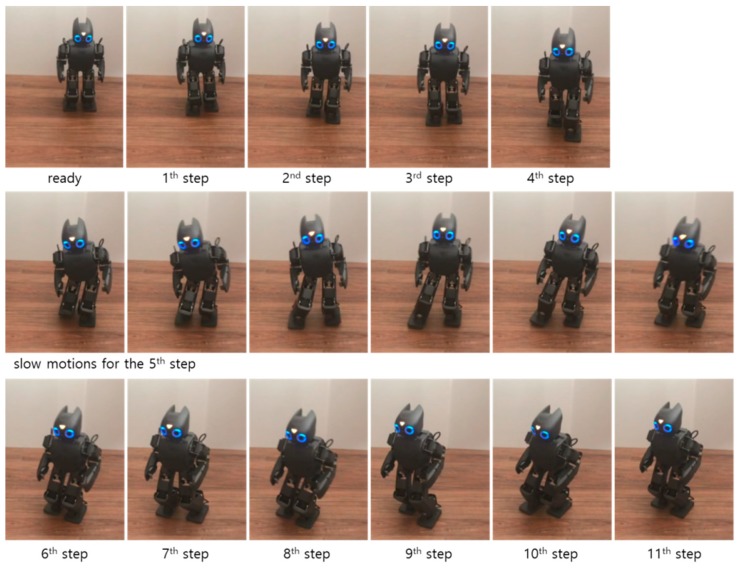
Snapshots of the experiment result without the walking stability controller (left to right, top to bottom).

**Figure 15:**
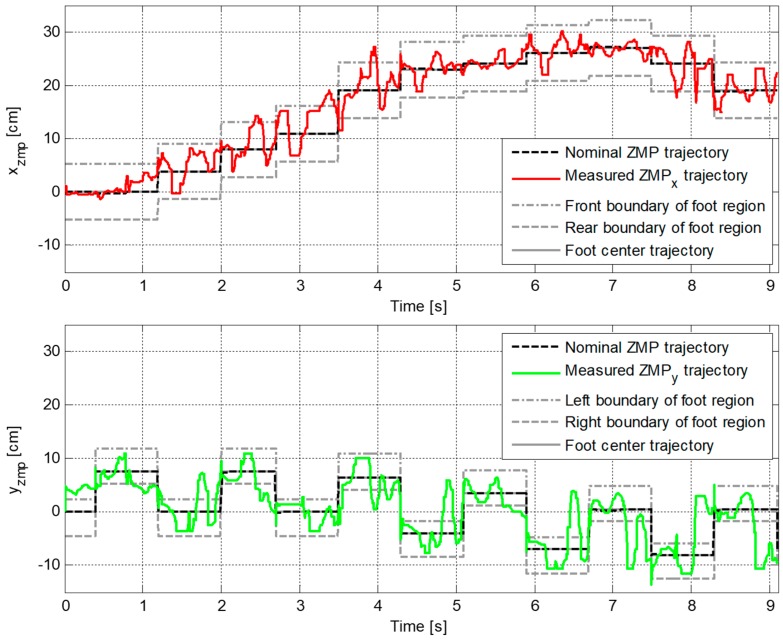
Trajectories of nominal ZMP obtained from the ZMP functions and measured ZMP, $p_{c}$, in the experiment without the walking stability controller.

Experiments were performed using a real bipedal robot, DARwIn-OP. The same footstep commands used in the first simulation were realized for the experiment on modifiable bipedal walking. A walking experiment that did not use the walking stability controller was performed first for the comparisons with the walking experiment that used the proposed walking stability controller. Figure 14 illustrates the snapshots of the experiment result without the walking stability controller. The robot walked unstably and almost fell as it stumbled at the fifth footstep, as shown in the Figure. In this experiment, CP tracking control was not performed, meaning the desired ZMP trajectory for CP tracking control, $p_{d}$, was not obtained. Accordingly, the measured ZMP trajectory was compared to the nominal ZMP trajectory obtained from the ZMP functions of the MWPG relative to the global coordinate system, as shown in Figure 15. The figure indicates that the measured ZMP trajectory followed the nominal ZMP trajectory with significant variation, and it was sometimes saturated. This instability was caused by the dynamic COM motion from the MWPG needed to perform the given footstep commands. Consequently, it was confirmed that a walking stability controller was required to successfully realize stable modifiable bipedal walking, including a sudden adjustment of foot placement during the single support phase.

**Figure 16:**
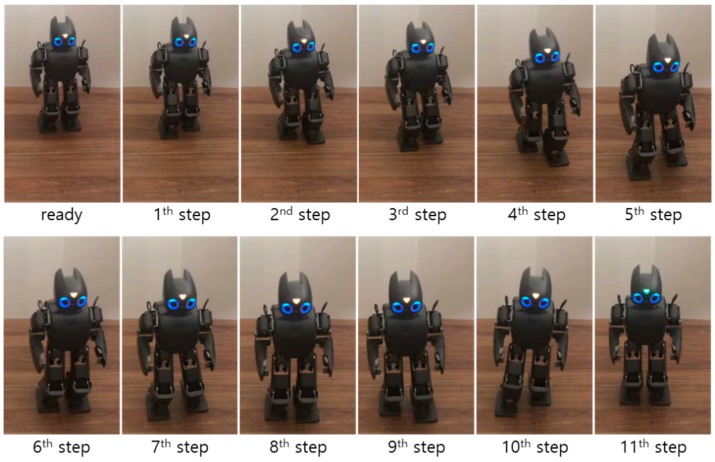
Snapshots of the experiment result with the proposed CP-based walking stability controller (left to right, top to bottom).

**Figure 17:**
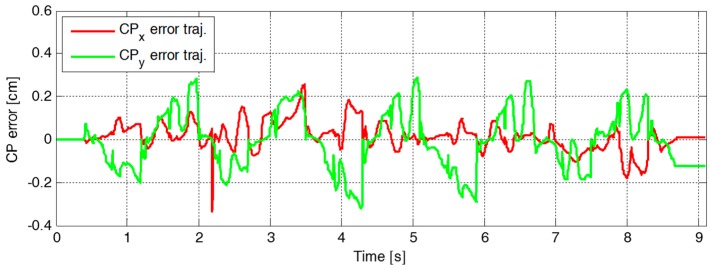
Error trajectory between reference and measured CPs, $\xi_{ref}$ and $\xi_{c}$, in the experiment with the proposed CP-based walking stability controller.

**Figure 18:**
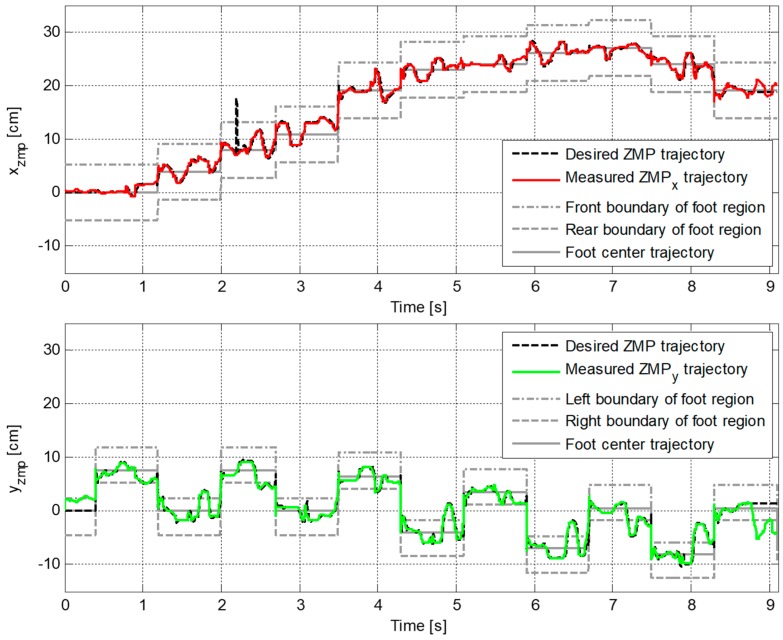
Trajectories of the desired ZMP for CP tracking control and measured ZMP, $p_{d}$ and $p_{c}$, in the experiment with the proposed CP-based walking stability controller.

A second walking experiment was then performed using the proposed CP-based walking stability controller, as shown in Figure 16. Figure 17 illustrates the error trajectory between reference and measured CPs, $\xi_{ref}$ and $\xi_{c}$, in the experiment with the proposed CP-based walking stability controller. The real CP tracked the reference CP trajectory with a little error. The error between the reference and the measured CP trajectories was larger than in the simulation results. This occurred because of measurement noise, offsets, and unmodeled errors in the real robot, including joint compliance. Accordingly, the desired ZMP trajectory for CP tracking control was considerably perturbed to allow the real CP to track the reference trajectory, as shown in Figure 18. However, the ZMP successfully followed the desired trajectory by using the controller with a little variation from the foot center trajectory. Therefore, the real CP trajectory was able to follow the reference CP trajectory. Consequently, the robot was controlled to walk stably according to the dynamically modifiable walking patterns provided by the footstep commands.

## 5. Conclusions

This paper proposed a CP-based walking stability controller to allow a bipedal robot to walk stably with a dynamically modifiable walking pattern. Infeasible footstep commands input to the MWPG were modified into feasible footstep commands through the handling method. Then, a reference CP trajectory for the single support phase was generated in real-time through ZMP manipulation without information on future footstep commands. This trajectory could be changed at any time according to the given footstep commands, which facilitated CP-based walking stability control to respond to sudden changes in navigational commands during the single support phase. The reference CP trajectory during the double support phase was also generated for continuity, and the CP of the robot was controlled to track the reference trajectory. The effectiveness of the proposed method was demonstrated through simulations and experiments.
